# Stalling chromophore synthesis of the fluorescent protein Venus reveals the molecular basis of the final oxidation step†

**DOI:** 10.1039/d0sc06693a

**Published:** 2021-03-31

**Authors:** Husam Sabah Auhim, Bella L. Grigorenko, Tessa K. Harris, Ozan E. Aksakal, Igor V. Polyakov, Colin Berry, Gabriel dos Passos Gomes, Igor V. Alabugin, Pierre J. Rizkallah, Alexander V. Nemukhin, D. Dafydd Jones

**Affiliations:** School of Biosciences, Molecular Biosciences Division, Cardiff University Sir Martin Evans Building Cardiff CF10 3AX UK jonesdd@cardiff.ac.uk +44 (0)29 2087 4290; Department of Biology, College of Science, University of Baghdad Baghdad Iraq; Chemistry Department, Lomonosov Moscow State University Leninskie Gory, 1-3 Moscow Russian Federation anem@lcc.chem.msu.ru +7 495 939 1096; Emanuel Institute of Biochemical Physics, Russian Academy of Sciences Moscow Russian Federation; Department of Chemistry, University of Toronto 80 St. George Street Toronto ON M5S 3H6 Canada; Department of Computer Science, University of Toronto 214 College St. Toronto Ontario M5T 3A1 Canada; Department of Chemistry and Biochemistry, Florida State University Tallahassee Fl 32306 USA alabugin@chem.fsu.edu +1 850 644 5795; School of Medicine, Cardiff University CF14 4XN UK

## Abstract

Fluorescent proteins (FPs) have revolutionised the life sciences, but the mechanism of chromophore maturation is still not fully understood. Here we show that incorporation of a photo-responsive non-canonical amino acid within the chromophore stalls maturation of Venus, a yellow FP, at an intermediate stage; a crystal structure indicates the presence of O_2_ located above a dehydrated enolate form of the imidazolone ring, close to the strictly conserved Gly67 that occupies a twisted conformation. His148 adopts an “open” conformation so forming a channel that allows O_2_ access to the immature chromophore. Absorbance spectroscopy supported by QM/MM simulations suggests that the first oxidation step involves formation of a hydroperoxyl intermediate in conjunction with dehydrogenation of the methylene bridge. A fully conjugated mature chromophore is formed through release of H_2_O_2_, both *in vitro* and *in vivo*. The possibility of interrupting and photochemically restarting chromophore maturation and the mechanistic insights open up new approaches for engineering optically controlled fluorescent proteins.

## Abstract 2

Fluorescent proteins (FPs) have revolutionised the life sciences, but the chromophore maturation mechanism is still not fully understood. Here we photochemically trap maturation at a crucial stage and structurally characterise the intermediate.

## Graphical abstract

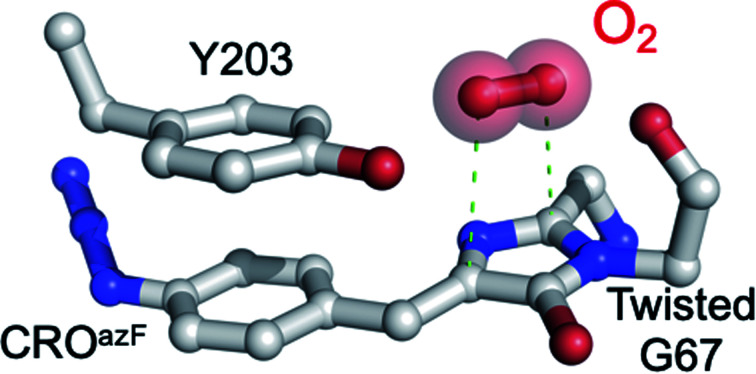

## Introduction

**Fig. 1:**
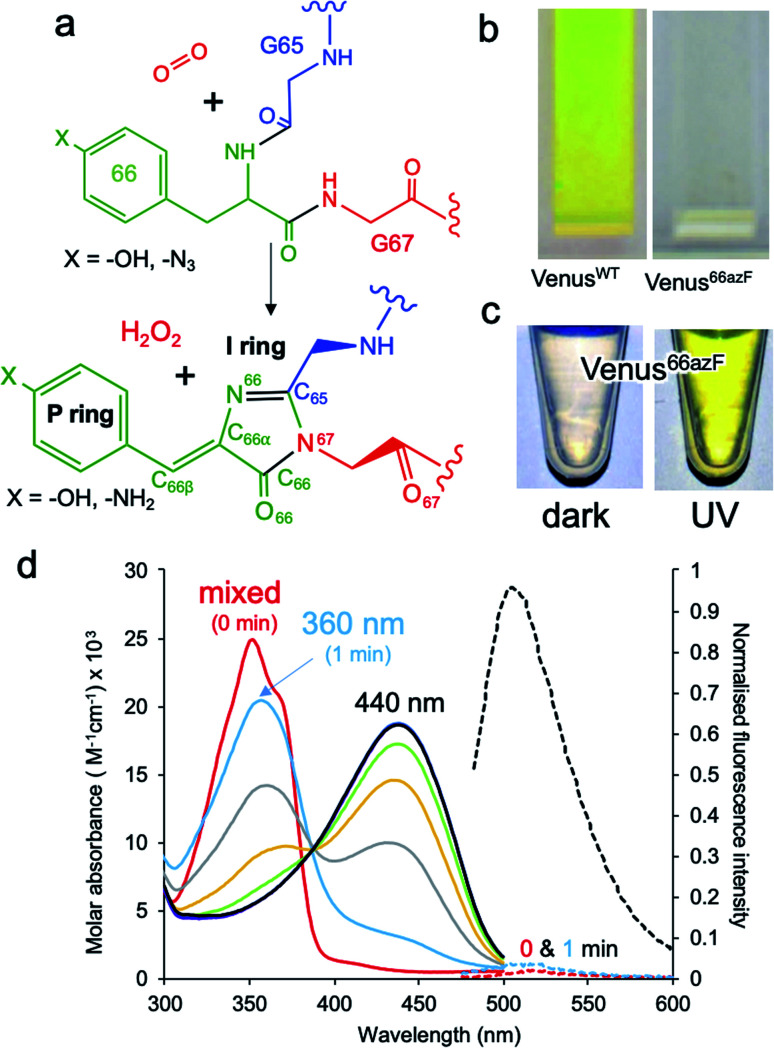
Effect of azF incorporation at residue 66 of Venus. (a) Scheme outlining the basic maturation of Venus. The bond and ring nomenclature are described in the lower structure. (b) Solution colours of Venus^WT^ and Venus^66azF^. (c) Solution colours of Venus^66azF^ before (dark) and after (UV) illumination with UV light. (d) Absorbance (solid line) and fluorescence (dashed line) of Venus^66azF^. Red, light blue, grey, orange, green, purple, dark blue represent spectra after 0, 1, 5, 10, 15, 30, 45 and 60 min. Full spectral properties are shown in ESI Table S1.† Full fluorescence emission time course and *in vivo* imaging are shown in ESI Fig. S1.†

Fluorescent proteins (FPs) represent an important family of proteins that emit light in the visible region of the spectrum without the requirement of any additional cofactor.^1–4^ Their unique fluorescence properties have revolutionised the life sciences through their use as genetically encoded imaging probes and sensors.^5,6^*In situ* synthesis of the chromophore is critical and occurs through the stepwise covalent arrangement of three contiguous residues Xaa-Tyr-Gly (where Xaa can be various amino acid residues), with only molecular O_2_ needed (Fig. 1a). In the case of Venus, these three residues are 65-Gly-Tyr-Gly-67 (Fig. 1a). Venus is a yellow fluorescent derivative of the classical *Aequorea victoria* GFP protein.^7^ Amongst the various mutations introduced to generate this yellow variant, two key changes are the introduction of glycine at residue 65 (from serine), which is thought to influence chromophore flexibility,^8^ and tyrosine at residue 203 (from threonine); Tyr203 π-stacks with the chromophore causing a red shift in excitation and emission into the yellow region.^7,9^ The mature chromophore has an extended conjugated bond network comprised of the phenolic P ring and the imidazolone I ring linked *via* a methylene bridge (see Fig. 1a for ring and atom notation), which is located within the core of the β-barrel structure.^1,4,10,11^ The first step in chromophore formation is cyclisation through linkage of the backbone amine of the strictly conserved Gly67 residue to the carbonyl carbon of the Xaa 65 residue. Cyclisation is followed by the dehydration/O_2_-dependent oxidation steps to generate a fully conjugated system. There has been much discussion about the exact mechanism of chromophore formation, especially the order of the dehydration and dehydrogenation/oxidation steps,^4,8,10–12^ and the role O_2_ plays in the rate-limiting oxidation step.^13^ The availably of structures representing trapped intermediates has helped shed some light on potential mechanisms^14–19^ but the exact role O_2_ plays in the maturation process still needs to be fully addressed. This is in part hampered by neither the crucial O_2_-bound form nor the oxygenated intermediates being experimentally observed. The former is especially critical as it can act as a realistic starting point for theoretical simulations. Gly67 is another key residue that is critical to chromophore synthesis and is strictly conserved amongst fluorescent proteins,^17,20^ but its role in maturation is unclear.

Manipulating the chemical properties of the chromophore by protein engineering, either directly through changes to two of the three chromophore residues, or indirectly through changing the chromophore environment, have generated a range of new fluorescent proteins, including Venus itself,^7^ with properties suited to their particular application.^6^ One of the most important FP class for super-resolution imaging is the photo-controllable FPs, whereby fluorescence is either switched on/off, or spectral properties significantly shifted in response to light.^21–24^ Mechanisms of action involves chemical modifications such as decarboxylation of Glu222 (*e.g.* PA-GFP^25^), backbone cleavage (*e.g.* Kaede^26^) and chromophore hydration (*e.g.* Dreiklang^27^), or conformational changes such as chromophore *cis*/*trans* isomerisation (*e.g.* Dronpa,^28^ rsEGFP^29^). The use of photochemically active non-canonical amino acids (ncAA) has further expanded optical control approaches.^30^ Phenyl azide photochemistry is particularly useful as we have used it previously to turn on, off, or switch the fluorescence properties of green^31,32^ and red^33^ FP types. Replacement of the chromophore forming tyrosine residue in super-folding GFP (sfGFP)^32^ and mCherry^33^ is known to impede fluorescence until irradiated; the azide group is thought to act as an excited-state quencher until converted to the amine.

Here we use the photochemical properties of genetically encoded phenyl azide to stall Venus chromophore maturation at an immature non-fluorescent intermediate (termed im-Venus^66azF^) state before UV irradiation instigates maturation to a final fluorescent form. The structure of the intermediate reveals the protein has undergone the dehydration but not the oxidation step. Additional new structural features add further new insights, including an essential role for the strictly conserved Gly67 and, for the first time, experimental observation of a putative molecular O_2_ in direct proximity to an enolate form of the dehydrated immature chromophore. The combination of experimental spectroscopy with quantum mechanics/molecular mechanics (QM/MM) simulations allowed us to propose a mechanism for the O_2_ dependent oxidation step whereby a hydroperoxyl intermediate is formed as part of the oxidation mechanism.

## Results

### Incorporation of phenyl azide chemistry within the Venus chromophore

To incorporate a photochemical switch into Venus, we replaced the chromophore residue Tyr66 with the aromatic non-canonical amino acid *p*-azido-l-phenylalanine (azF);^34,35^ effectively the hydroxyl group of tyrosine is replaced with an azide group. The variant termed Venus^66azF^ is produced as a colourless protein (Fig. 1b) with no inherent fluorescence (Fig. 1b–d and S1†), and acts an excellent starting point for an optically controlled FP. Mass spectrometry confirmed that azF was incorporated and that the chromophore had at least gone through the cyclisation–dehydration step (Fig. S2†). The absorbance spectrum of the colourless non-fluorescent Venus^66azF^ reveals the protein absorbs in the UV region. Absorbance peaks at ∼350 nm (∼3.5 eV) with a significant shoulder at ∼360–370 nm (∼3.44–3.35 eV); no fluorescence is observed from this species (Fig. 1d and S1a, b†). Irradiation of Venus^66azF^ with low intensity (6 W from a handheld lamp) near-UV light converts the protein to a coloured and fluorescent form both *in vitro* (Fig. 1c, d and S1a, b†) and *in vivo* (Fig. S1c and d†), with an absorbance *λ*_max_ shifting to a broad 440 nm peak (2.82 eV) (ESI Table S1†), similar to that observed previously for the amine chromophore product in sfGFP.^32^ Fluorescence emission increased over 100-fold on activation, allowing imaging of bacterial cells expressing the activated Venus^66azF^ (Fig. S1d†). Spectroscopic analysis of the time course of activation reveals a potential two-step process. Within 1 min of UV exposure, the double hump absorbance spectrum feature disappears, with the remainder of the time points forming a clear isosbestic point at ∼390 nm (3.18 eV) suggesting conversion directly from one form to another. As the initial dark sample diverges away from the isosbestic point, it suggests a primary conversion to an intermediate species, which is also non-fluorescent, followed by a slower conversion to the activated form. The final product of UV irradiation was the phenyl amine version of the chromophore, as observed previously for sfGFP^66azF^,^32^ but it appeared sensitive to fragmentation on denaturation due to cleavage close to the chromophore (see ESI Fig. S2† and associated discussion); fragmentation within the chromophore on denaturation is commonly observed for GFP-like proteins.^24,36^ SDS-PAGE analysis of the equivalent UV timepoints suggests that UV irradiation does not generate a completely fragmented protein (ESI Fig. 2†); after 5 min fragmentation remains relatively constant (20–25%), in line with previous denaturation-dependent fragmentation observations.^24,36^ The mass spectrometry analysis did not indicate a secondary decarboxylation (*e.g.* Glu222) event that can sometimes be associated with UV irradiation of fluorescent proteins.^37^ UV irradiation of Venus^WT^ did not result in any spectral shifts (ESI Fig. S1e and f†).

### Structure of im-Venus^66azF^

**Fig. 2:**
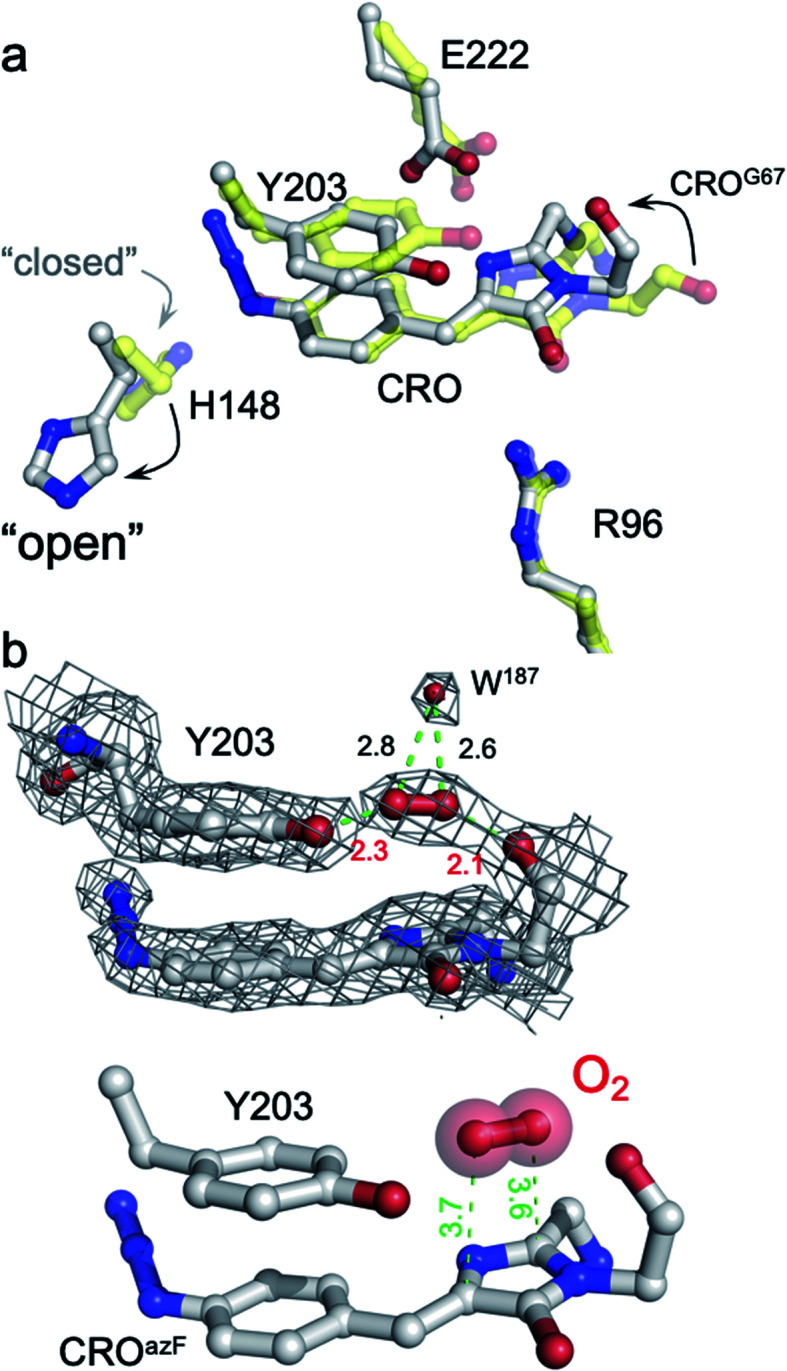
Structure of im-Venus^66azF^ proximal to the chromophore. (a) Comparison of im-Venus^66azF^ (grey; PDB 6sm0) with Venus (yellow; PDB 1mwy^9^). CRO is the chromophore (Gly65-Tyr/AzF66-Gly67). (b) Position of the O_2_ molecule in im-Venus^66azF^. The top panel shows the electron density (2Fo-Fc, 1.0*σ*) for the CRO, Y203 and O_2_ together with an additional water molecule. The lower panel removes the electron density for clarity. Relevant distances are shown in Å.

The structure of the pre-photoactivated Venus^66azF^ (from herein referred to as immature or im-Venus^66azF^) was determined to 1.9 Å resolution (see ESI Table S2† for structural statistics) to gain an insight into the molecular nature of the non-fluorescent, immature chromophore. The general structure of im-Venus^66azF^ is similar to mature wild-type Venus (henceforth termed Venus^WT^), with a root-mean-squared deviation (RMSD) over the backbone of 0.39 Å (ESI Fig. S3a†). The most significant differences occur in and around the chromophore (termed CRO). Electron density fits well to a cyclised chromophore containing the intact azide group present on the P ring (Fig. 2 and S3b†). The lack of the electron density protruding from the I ring at the C_65_ position suggests the dehydration step in chromophore maturation has occurred by this point (ESI Fig. S3b and c†). Compared to Venus^WT^, the I ring component shifts position in im-Venus^66azF^ due to the more acute angle in the methylene bridge (Fig. S4b†). A comparison of the residues surrounding the CRO shows that several residues exist in different conformations compared to Venus (Fig. S4†). His148, a critical H-bonding residue to the mature CRO, exists fully in the open-gate conformation. Glu222, another residue necessary for function, and Tyr203 (π-stacks with the CRO in Venus^WT^) also shift position with respect to the CRO (Fig. 2a). The newly introduced phenyl azide group occupies a similar position as the tyrosyl residue in Venus (Fig. 2a and S4†). The azide moiety itself is located between Gln204-Ser205 and Tyr145-Ser147, displacing a water molecule normally present in Venus, with Ser205 hydroxyl group making potential polar interactions with the azide.

Further analysis of the chromophore, including comparative estimates of bond distances between the refined structure of im-Venus^66azF^ with the refined Venus structure, reveals several novel features that provide us with insights into CRO maturation and the role of molecular O_2_ in the process. In im-Venus^66azF^, the backbone carbonyl of the strictly conserved Gly67 is twisted ∼180° out of position compared to that observed in other FPs (Fig. 2a, b and ESI Fig. 3d, e†). To our knowledge, the only other time this conformation has been observed is in the unpublished structure of an immature chromophore of a GFP maturation disabling mutant determined by the Getzoff group (PDB 2qt2; ESI Fig. S5†). There is additional electron density sandwiched between the Gly67 carbonyl group and the hydroxyl group of Tyr203 (Fig. 2b and ESI Fig. 3d and e†), which we have assigned to molecular O_2_ after attempting to refine the structure with either one or two H_2_O. We found the presence of Tyr203 effectively blocks the ability of two water molecules to occupy this position whereas one water molecule left an elongated tail of density. We found that molecular O_2_ fitted best; the difference map is featureless around the elongated density fitted to O_2_ with no positive (no atoms unaccounted for) nor negative difference (atoms that should not be where they are modelled), as can be seen in Fig. 2b. The O_2_ molecule lies between the twisted carbonyl oxygen of the Gly67 and the hydroxyl group of Tyr203 above the plane of I ring element (Fig. 2b). O_2_ has been postulated to be positioned either above the plane of the chromophore facing Glu222/Tyr203 or below the chromophore plane facing Arg96;^18,38^ here O_2_ is above the plane of the chromophore on the Glu222/Tyr203 face (Fig. 2b).

**Fig. 3:**
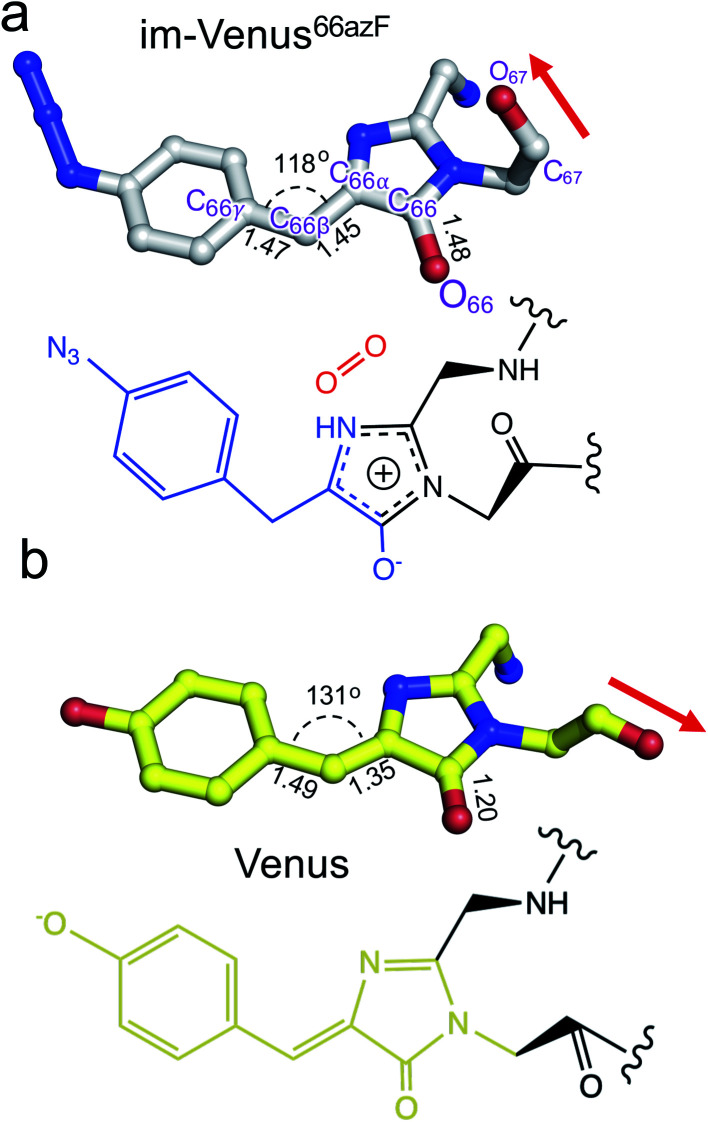
Chromophore structures of im-Venus^66azF^ and mature Venus^WT^ fluorescent proteins. (a) im-Venus^66azF^ with proposed chemical structure; (b) Venus^WT^ (PDB code 1myw^9^) with the chemical structure. A comparative analysis of the selected chemical bond lengths is shown.

The structure also provides an insight into the nature of the trapped chromophore intermediate (Fig. 3). During maturation, O_2_ is thought to be involved in generating the final C=C that links the P-ring and I ring (C_66β_–C_66α_ to C_66β_=C_66α_). Comparative analysis of the C_66β_–C_66α_ bond lengths in the refined structure suggests it could be a single bond in im-Venus^66azF^ and a double bond in Venus (Fig. 3). The bond angle between C_66γ_–C_66β_–C_66α_ is also more acute for im-Venus^66azF^ (118° *versus* 131° for Venus; Fig. 3). In the I-ring, the C_66_–O_66_ bond is 1.48 Å for im-Venus^66azF^, which is longer than would be expected for a keto-carbonyl C=O bond (1.20 Å) as observed in Venus (Fig. 3b). This longer C–O bond also makes polar contacts with the critical maturation residue Arg96, which occupies a near-identical position in Venus^WT^ (Fig. 2a). Thus, we predict that the enolate is the most likely form of C_66_–O_66_ (Fig. 3a) with Arg96 stabilising the negative charge. The negative charge on the enolate can be offset by a positive charge delocalised around the I ring, as proposed in Fig. 3a.

### Simulation of the oxidative step of chromophore maturation

To correlate the structural and functional observations, modelling of reaction intermediates was performed using the atomic coordinates of im-Venus^66azF^ obtained in this work as a starting point. Structures of potential intermediates were assumed on the basis of previous experience in modelling chromophore maturation in GFP^38^ and optimized in QM/MM calculations. Analysis of the obtained results for Venus^66azF^ shows that mechanistic steps during the oxidation step of chromophore maturation are essentially the same as in the wild-type GFP.^38^

**Fig. 4:**
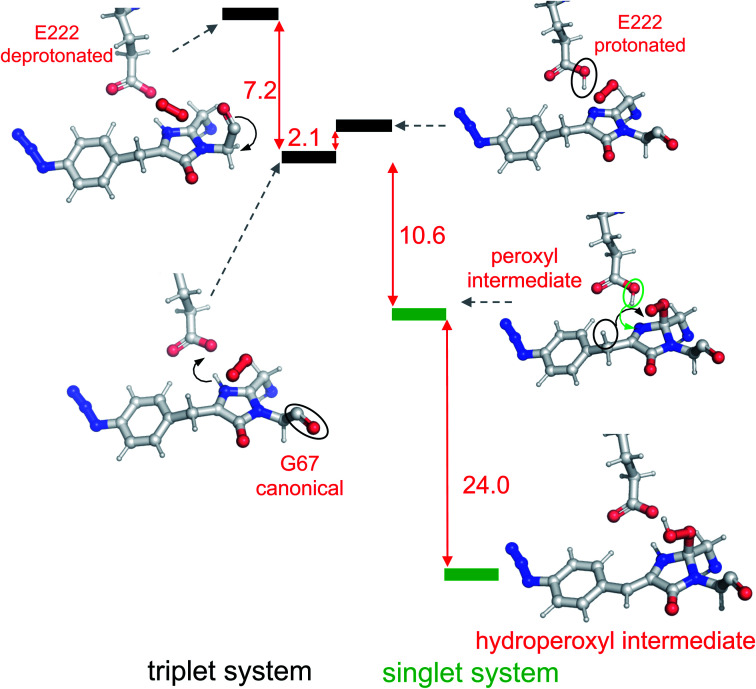
Reaction scheme for oxygenation of the chromophore based on modelling results. The panels show relevant segments of the assumed protein structures optimized in QM/MM calculations. Triplet and singlet systems are differentiated by black and green lines, as indicated in the diagram. The Δ*E* between each state is shown in red with units of kcal mol^−1^. Significant changes between each state are outlined in the diagram.

**Fig. 5:**
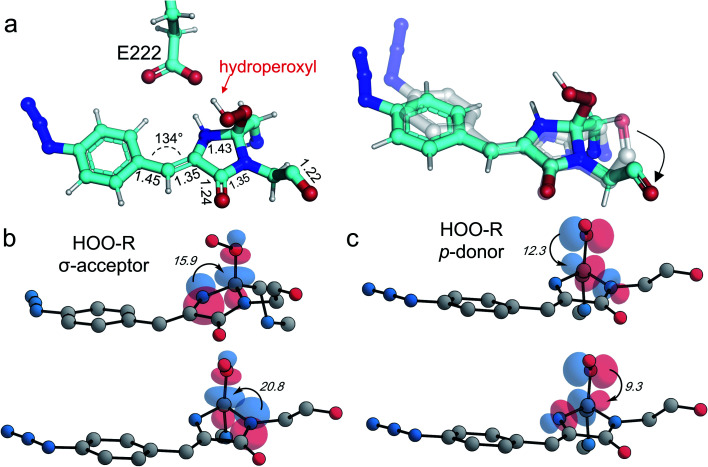
Models of the hydroperoxyl intermediate. (a) Model structure as determined by QM/MM simulations with the hydroperoxyl group bound to C_65_ as indicated. On the right-hand side is a comparison of the modelled hydroperoxyl intermediate (cyan) with the crystal structure of im-Venus^66azF^. (b and c) Stereoelectronic interactions in hydroperoxyl intermediate (hydrogen atoms omitted for clarity with the numbers related to energies in kcal mol^−1^). Both the hydroperoxyl moiety acting as a σ-acceptor (b) and p-donor (c) are shown.

The overall scheme based on the simulations is shown in Fig. 4, and the corresponding structures were associated with the observed absorbance data. The simulations show that the first step involves Gly67 switching to its energetically more favourable (∼7 kcal mol^−1^) canonical configuration. Thus, it appears we were fortunate that the crystalline form of im-Venus^66azF^ was trapped in the observed conformation shown above. Triplet state oxygen can now access the I ring with concomitant protonation of Glu222, which acts as a general acid/base in the maturation scheme. The oxidation steps then proceed starting from partial negative charge transfer to O_2_, which switches from the triplet to singlet state. Glu222 is protonated with N_65_ donating the proton (Fig. S6†). The O_2_ then attacks C_65_ (and not C_66α_) generating a peroxy intermediate (Fig. 4 and S5†). The peroxy anion then abstracts a proton from C_66β_ to form the stable hydroperoxyl intermediate with Glu222 protonating N_65_ (Fig. 4 and S6†); the hydroperoxyl species has a theoretical absorbance of 360 nm (3.44 eV). As well as Gly67 converting through to its canonical conformation, the formation of the methylene bridge between the I and P rings results in a shift to a configuration similar to that observed for the mature chromophore observed in Venus^WT^; the methylene bridge bond angle is now 134° (Fig. 5a), similar to that observed for Venus^WT^ (Fig. 3b).

We also undertook natural bond orbital (NBO) analysis to evaluate the hydroperoxyl intermediate. NBO analysis transforms electron density from DFT calculations into localized orbitals that are closely tied to the chemical bonding concepts. In particular, NBO analysis is commonly used to evaluate hyperconjugative stabilising interactions.^39,40^ The lone pair of the hydroperoxyl group (–OOH) in the model has near-ideal alignment with the two C–N bonds (Fig. 5b and c). This favourable stereoelectronic arrangement activates stabilizing hyperconjugative 
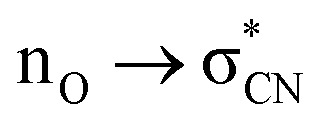
 interactions, which can partially compensate for the loss of aromatic stabilisation in the I ring. This is complemented by two strong 
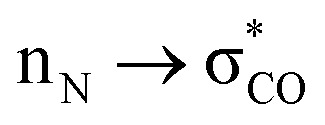
 interactions. The importance of the latter effect is expected to grow further in the transition state for the final C–O bond scission where it provides an important transition state stabilization effect that can significantly assist the final aromatizing step of the cascade.^41–43^

**Fig. 6:**
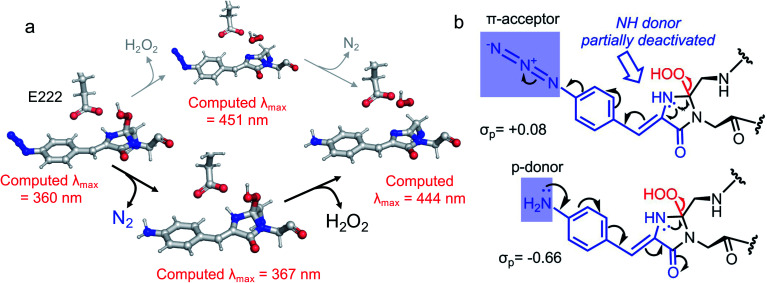
Conversion of phenyl azide intermediate to mature chromophore. (a) Potential routes to mature phenyl amine chromophore from the hydroperoxyl intermediate. The two routes are azide reduction followed by full chromophore conjugation (black route) and *vice versa* (grey route). The theoretical *λ*_max_ values are shown. It should be pointed out that the presumed species without the hydroperoxyl and phenyl azide present (*λ*_max_ = 451 nm, 2.75 eV) is considerably higher in energy than the hydroperoxyl intermediate. Therefore, the upper grey route is unlikely, as also clarified in (b). (b) Mechanistic analysis highlights the contrasting effects of azide and amine substituents on the elimination of hydroperoxide in the final aromatisation step. The decelerating effect of the acceptor azide on the OOH elimination is deactivated once the azide is converted into a strongly donating amine moiety.

The final step occurring over the irradiation period is the conversion to a fully mature fluorescent chromophore. Two events need to be considered: reduction of the azide and full conjugation of the chromophore through a loss of the hydroperoxyl moiety. The spectral properties suggest that the final end product is likely to be the phenyl-amine form of the mature chromophore as has been observed before in superfolder version of GFP (sfGFP; Table S1† and ref. 32); this was confirmed by mass spectrometry (ESI Fig. S2†). Simulations concur with this with the final product having a theoretical absorbance max at 444 nm (2.79 eV), close to the 440 nm (2.82 eV) observed in Fig. 1d. Full chromophore conjugation with the azide left intact will generate a species less stable than the preceding step and has a theoretical absorbance maximum at 451 nm (2.75 eV) (Fig. 6a). The alternative route appears more likely: reduction to the phenylamine followed by loss of the hydroperoxyl group (generating H_2_O_2_). The phenylamine version of hydroperoxyl intermediate has a theoretical absorbance maximum of 367 nm (3.38 eV). Based on the experimental time course observed in the Fig. 1d, the initial species mix dominated by the hydroperoxyl intermediate (computed *λ*_max_ = 360 nm, 3.44 eV) within 1 min of irradiation converts to the dominant hydroperoxyl phenylamine (computed *λ*_max_ = 367 nm, 3.38 eV) that then forms the mature phenylamine chromophore.

### Oxygen access to the chromophore and the twisted Gly67

**Fig. 7:**
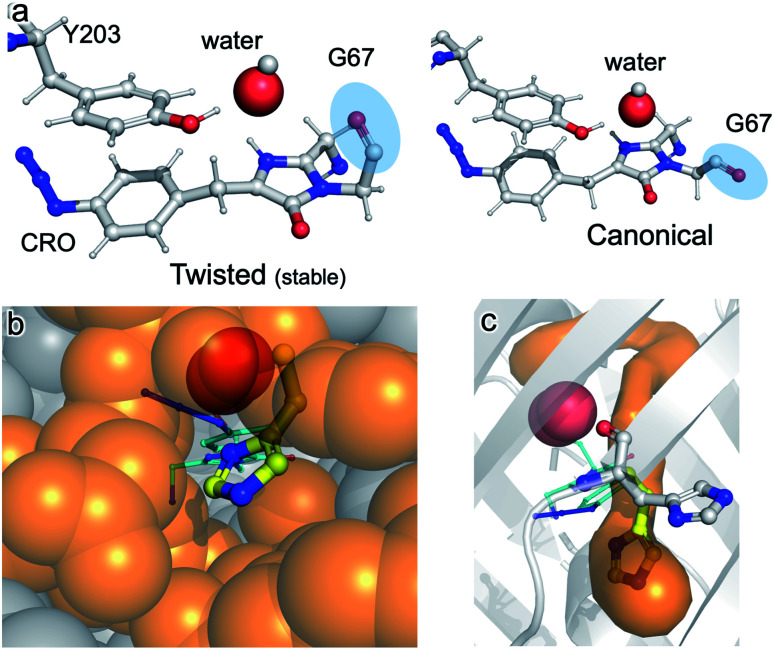
Role of water and internal tunnels in chromophore maturation. (a) Simulation of the twisted conformation stabilised by a water molecule in place of O_2_ (and for reference, the canonical form usually observed in mature Venus^WT^ conformation). The twisted form is more stable by 18.1 kcal mol^−1^ compared to the canonical form. The water molecule of interest is highlighted. Chromophore accessibility, as shown by (b) spheres and (c) CAVER tunnel analysis.^44^ The alternative conformation for His148 (yellow sticks) in Venus^WT^ is shown.

The structure of im-Venus^66azF^ revealed the presence of O_2_ within the core of the protein close to the I ring of the chromophore. There are two potential ways in which O_2_ becomes located in such a position: during the folding process or diffusion into the core after folding to the β-can structure and initial chromophore cyclisation. Simulations, whereby O_2_ is replaced by a water molecule, reveal that the twisted conformation of the immature chromophore is more stable by 18.1 kcal mol^−1^ than the canonical form (Fig. 7a). The presence of water is unsurprising given that O_2_ is not required for the preceding cyclisation and dehydration events. Thus, water helps stabilise the twisted form of Gly67 present in im-Venus^66azF^. If this is the case, O_2_ is likely to displace the water molecule as part of the maturation mechanism, meaning it has to gain access to the core of the protein. Analysis of the im-Venus^66azF^ structure reveals that the “open” His148 conformation may play a role (Fig. 7b and c). A channel leads to the chromophore in im-Venus^66azF^ that is only available if His148 occupies the “open” state; when His148 occupies the “closed” state normally observed in mature fluorescent proteins, the tunnel becomes blocked.

## Discussion

Photo-controllable FPs have become an important tool in modern super-resolution cell imaging.^45^ The use of phenyl azide chemistry to control fluorescence, here and in green^31,32^ and red^33^ fluorescent proteins, provides a simple and general common mechanism to implement photocontrol across a broad range of the FP colour palette. Indeed, the use of a ncAA in conjunction with a reprogrammed amber stop codon allows secondary control: production of the fusion protein constructs with and without the FP adjunct.^46^ While the demonstration of the conversion of an essentially colourless immature fluorescent protein to an active form through phenylazide photo-decaging confirms the approach as a means to photo-control FPs, insights into the chromophore maturation process are arguably the most important aspect of this work.

**Scheme 1:**
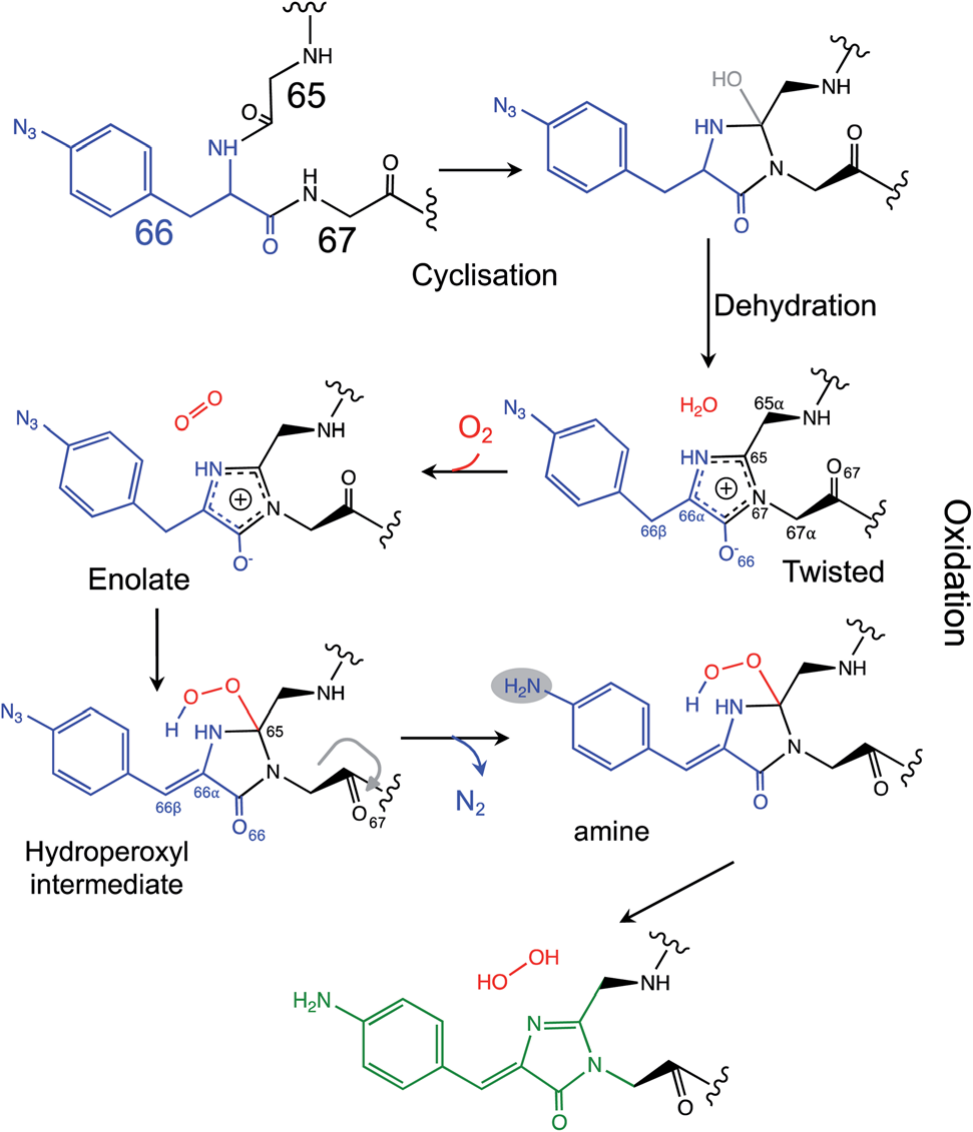
Proposed mechanism for the chromophore maturation, including details of the final oxidation step.

Venus is an engineered version of the original *Aequorea victoria* GFP, that includes mutations that comprise the chromophore (S65G) and directly interact with the chromophore (*e.g.* T203Y) so contributing towards its red-shifted fluorescence properties. There are currently two models that describe the overarching process by which chromophore maturation in *A. victoria* derived fluorescent proteins occur:^4^ “cyclisation–oxidation–dehydration”^12,15^ and “cyclisation–dehydration–oxidation”^13,18,47^ both of which are supported by experimental data albeit through using different approaches. Our data supports the “cyclisation–dehydration–oxidation model, at least in the context of Venus^66azF^ and under the conditions we used. As has been pointed out previously, both maturation processes may indeed occur in parallel with factors such as oxygen concentration and local mutational events influencing the order.^4^ In our study, the experimentally obtained trapped intermediate structure of im-Venus^66azF^ provided evidence that dehydration had already taken place (Fig. S3†) but oxidation had yet to occur (Fig. 3). Coupled with the simulation data, we propose a chromophore synthesis route for Venus^66azF^ outlined in Scheme 1.

The crystal structure of the im-Venus^66azF^ provides evidence for the nature of an intermediate prior to the final oxidation step: the I ring in the enolate form with the C_66α_ and C_66β_ forming a single bond (Fig. 3a). The long C_66_–O_66_ bond length is indicative of the enolate whose negative charge is stabilised by critical chromophore maturation residue, Arg96 (Fig. 2a).^16,20,48^ The crystal structure also provides evidence of the location of O_2_. Two relative positions with respect to the chromophore plane have been proposed: on the Arg96 (ref. 18) or the Glu222 face.^38^ Here we show that in our system molecular oxygen is placed on the Glu222 face, directly above the I ring (Fig. 2), in agreement with recent simulation data for GFP.^38^ Arg96 has been suggested as the oxygen activator through the positively charged side-chain^18^ but this is unlikely to be the case here and may instead play a role in stabilising the enolate form of the I ring.

The twisted Gly67 configuration is clearly observed in the crystal structure and differs from the canonical position normally present in FPs (Fig. 2). Glycine has a less restricted ψ angle range. In Venus, the Gly67 ψ dihedral angle is −23° compared to 167° in im-Venus^66azF^, with twisted conformation energetically less favourable (by 7 kcal mol^−1^) when O_2_ is present and also hinders access of oxygen to the I ring (Fig. 2). The twisted conformation could be a legacy of the cyclisation reaction whereby the nucleophilic attack of N_67_ on C_65_ will require rotation of the Gly67 ψ angle leading to its observed placement in im-Venus^66azF^. While we were fortunate to have trapped this immediate form in the crystalline state, the configuration could be stabilised by the O_67_ H-bonding with the C_65_ hydroxyl group (observed by Getzoff and colleagues^17^) before condensation. The simulation data also revealed that a single spatially defined water molecule can stabilise the twisted conformation over the canonical form more so than the O_2_ bound form (Fig. 5a) that our data suggests is likely be present in the crystal structure. This could suggest that a hydrated form precedes the oxygen bound step. It is interesting to speculate that the origin of the water molecule may be the product of the cyclisation/dehydration reaction that precedes oxidation. Indeed, while our crystal structure of im-Venus^66azF^ strongly indicates O_2_ is present, we cannot rule out that a population of the structures has a water molecule present in the same position: fitting the observed electron density to a dynamic water over two sites suggests this could be feasible. However, we feel the evidence for O_2_ being the main species is: (1) if the O in water takes the position of one or other O atoms in the O_2_ molecule, this will generate steric issues due to the presence of the H atoms; (2) the position of O_2_ in the structure correlates with the QM/MM work here and elsewhere^38^ and available tunnels through to the chromophore with H148 in the open configuration. The most likely scenario is that there is mixture of O_2_ and water occupying the site, with O_2_ occupancy likely being the dominant population observed in our crystal structure. It is interesting to speculate that the alternative conformation of G67 may induce a dipole in O_2_ due to the close proximity of the *δ*^−^ on O_67_, resulting in an interaction network spanning to Tyr203.

If a water molecule originally stabilised the twisted Gly67 conformation, this suggests that O_2_ needs to access the protein core for the final oxidation step to take place. We propose that His148 plays a key role in this process. His148 is dynamic^49^ and has been observed in both the “open” and “closed” conformations with the former not normally reported in the crystal structure as it is a minor component when observed (for example Arpino *et al.*^50^ Reddington *et al.*^32^ and Brejc *et al.*^51^); the closed conformation is the major form observed in mature FPs as His148 in this configuration H-bonds to the chromophore and plays a critical role in function.^1,10,52,53^ Im-Venus^66azF^ almost exclusively exists in the open conformation (Fig. 2a and S4†) that generates a channel through to the chromophore (Fig. 7b and c). Such a tunnel at a similar position has been observed previously for GFP-like proteins.^54,55^ In Dreikling, a reversible photo-switchable close relative of Venus, H148 exists predominantly in its “open” conformation that can potentially allow water access to the chromophore as part of the hydration event that underlies photochemical control.^27,56^ Thus, His148 may acts as a “gatekeeper” residue, so determining access to the chromophore as well as its functional role (H-bond to chromophore phenol group).^49^ Given that oxidation is the rate-limiting step in maturation,^4^ it is interesting to speculate that the exchange rate between the two His148 conformations may play a role in defining this rate.

Simulations suggest that the next dominant form is hydroperoxyl intermediate attached to C_65_ and not C_66α_, as suggested by others.^18^ The presence of a hydroperoxyl intermediate attached to C_65_ has been proposed previously for GFP.^38^ Computational modelling of GFP suggests that O_2_ may form a bridge between the C_65_–C_66α_ with a concerted proton transfer mechanism initially from C_66β_ resulting in the formation of the C_65_ attached hydroperoxyl intermediate and thus the β-methylene bridge.^38^ The argument against attachment to C_66α_ as the intermediate comes from the observed spectra data (Fig. 2b), whereby the dominant 350–360 nm peaks for the intermediate suggests some extension of the conjugated double bond system (here proposed to be from the phenyl azide to C_66α_–C_66β_; Fig. 5). The formation of a hydroperoxyl intermediate at C_66α_ would prevent the formation of the double bond with C_66β_. The electron lone pair on the C_65_ hydroperoxyl moiety also aligns perfectly with the two C–N bonds that, in turn, helps stabilise the five-membered I ring. We propose that the nearby Glu222 plays a vital role acting as a general acid/base during the formation of the hydroperoxyl intermediate through the first abstraction and then the donation of a proton to N_66_ (Fig. S6†). The importance of Glu222 to maturation has been observed previously, with the E222Q mutation in EGFP considerably slowing maturation.^20,48^ During this process we propose that a peroxyanion is formed, which abstracts the proton from the activated C_66β_ to form hydroperoxyl intermediate (Fig. 4 and S6†), as suggested for GFP.^38^ Thus, the formation of the C_66α_=C_66β_ double bond occurs before the generation of H_2_O_2_ and not concurrently (Scheme 1).

The proposed final and rate-limiting step in the process is the formation of the fully conjugated fluorescent chromophore. In our system, we believe this is a UV induced phase that happens in two steps due to the presence of the azido group: (1) conversion of the phenyl azide to the phenylamine; (2) loss of the hydroperoxyl group so generating a fully conjugated system. In our proposed model, we suggest that the reduction of the azide to an amine occurs first (Scheme 1). This is based on the computed theoretical absorbance of each species (Fig. 6a) and on the expectation that azide conversion to strongly donating amine group would significantly help with loss of the hydroperoxyl moiety (see Fig. 6b for mechanistic details). Furthermore, the departure of the OOH group needed for the conversion of hydroperoxyl intermediate to the final maturated chromophore is expected to be greatly facilitated when azide, a mild acceptor (Hammett parameter *σ*_p_ = +0.08), is changed to NH_2_, a strong donor (*σ*_p_ = −0.66). In the azide-substituted peroxide, the lone pair of N_66_ is not able to fully assist in the departure of the OOH group as its electron density is partially delocalized in the other direction, towards the azide. This stereo-electronic tug-of-war is removed once the amine is formed. As the NH_2_ group is a powerful donor, the electron density is no longer shifted from N_66_ to the aryl ring; the lone pair on N_66_ is now free to stabilize the transition state for heterolytic C⋯OOH bond scission. A fully conjugated chromophore with the azide group attached has a computed *λ*_max_ of 451 nm, whereas the amine version of hydroperoxyl intermediate is 367 nm. Given the observed absorbance time course in Fig. 2 goes from a mixed species with two peaks between 340–360 nm to a single species at 360 nm that directly converts to the single species at 440 nm, the logical progression of the computed spectra are 360 nm (phenyl azide/hydroperoxyl form) to 367 nm (phenylamine/hydroperoxyl forms) to 444 nm (mature amine chromophore). UV light may also play a role beyond the initial conversion from the azide to the amine as prolonged exposure is needed to fully develop fluorescence (Fig. S1† and ref. 30). From comparison of the absorbance and fluorescence time course, the initial intermediate observed after 1 min is not itself significantly fluorescent nor is the initial species absorbing at 351 nm suggesting additional UV-induced events are needed for full fluorescence. It is not clear why the further input of UV light is needed but the final step from the hydroperoxyl intermediate requires energy to overcome the last reaction barrier involving proton transfer from the I ring to E222 as part of the process of H_2_O_2_ generation.^38^ Given that Venus^66azF^ remains largely intact on UV exposure (Fig. S2b†) and does not have a HYG chromophore motif, backbone fragmentation is unlikely to be the main end-point of activation as observed in green-to-red photoactive FPs such as Kaede^26^ and EosFP.^57^ While simulations provide strong evidence (Fig. 6) that conversion of the azide to the amine is the initial step in the process we cannot completely rule out that this step may happen later in the scheme.

## Conclusion

For the first time, our X-ray structure of im-Venus^66azF^ provides an experimental evidence concerning insight into the role played by oxygen in fluorescent protein chromophore maturation. The fortuitous trapping of oxygen between Tyr203 and an alternative conformation of the strictly conserved Gly67 before photo-decaging of azF66 was critical. From our structure and subsequent modelling, the cyclisation–dehydration–oxidation model mechanism is the likely route to chromophore maturation, at least for Venus^66azF^, but with new insights concerning the nature of the enolate intermediate together with mechanistic details of the O_2_-dependent steps. These new insights not only improve our fundamental understanding of chromophore maturation but may help aid our ability to generate improved fluorescent protein for future applications.

## Methods and materials

### Engineering, production and structure of Venus^66azF^

The generation of the mutant and subsequent recombinant production of Venus^66azF^ is outlined in the ESI† and based on a previously published procedure.^55^ Protein production and purification was carried out in the dark to prevent photolysis of azF. Crystallisation, diffraction data collection and structure determination of im-Venus^66azF^ was performed as outlined in the ESI.† A long, spiny crystal of im-Venus^66azF^ was observed after a few days in the A12 condition of the PACT premier TM HT-96 screen (0.01 M zinc chloride, 0.1 M sodium acetate, pH 5 and 20% w/v PEG 6000). Data were collected at the Diamond Light Source (Harwell, UK) at beamline I04-1. Data reduction was completed with XIA2 (ref. 58) using XDS. Data scaling, merging and analysis were completed with AIMLESS^59^ and TRUNCATE^60^ in the CCP4 Package. Structure solution was made *via* molecular replacement with PHASER^61^ using PDB entry 4J88, the structure of sfGFP^66azF^. The structure was refined with REFMAC5,^62^ using the standard restraints supplied with the program. The geometry restraints for the chromophore were derived from ideal geometry with JLIGAND,^63^ allowing REFMAC to determine weights automatically. COOT^64^ was used for graphical sessions to adjust the model to match the electron density maps. The crystallographic statistics are provided in ESI Table 2.†

### Absorbance and fluorescence spectroscopy

Absorbance spectra were recorded using a Cary 60 spectrophotometer (Agilent) in a 1 cm pathlength quartz cuvette. The molar absorbance coefficients were calculated by recording absorbance spectra of Venus samples with a known concentration (5–10 μM) and then extrapolated to 1 M using the Beer–Lambert law. Emission spectra were recorded using a Cary Eclipse fluorimeter (Varian) using a 5 mm × 5 mm QS quartz cuvette. Protein concentration of 0.5 μM for Venus^WT^ or 10 μM for Venus^66azF^ was used with a fixed scan rate of 120 nm min^−1^ with a 5 nm slit width. The excitation wavelength for Venus^WT^ and Venus^66azF^ were determined from their absorbance spectrum. Bacterial live cell imaging was performed as outlined in the ESI.†

### Photolysis of Venus^66azF^

Photolysis experiments were carried out using a UVM-57 Handheld UV lamp (6 W; 302 nm UV, UVP Cambridge, UK) and 1 cm pathlength quartz cuvette (Hellma), essentially as described previously.^31,54^ A maximum of 500 μl of protein sample (10 μM) was pipetted into a cuvette and exposed to the UV (302 nm) for the indicated periods of time at a distance of 1 cm. The absorbance spectra and emission spectra were recorded immediately afterwards, as described above.

### QM/MM simulations

Coordinates of heavy atoms of the im-Venus^66azF^ crystal structure were used to construct a full atom three-dimensional model system. Previous examples in quantum mechanics/molecular mechanics (QM/MM) simulations of the mechanism of chromophore maturation included the wild-type GFP,^38^ its Gly65–Gly66–Gly67 mutant,^65^ and the recent modeling of Dreiklang,^56,66^ a photo-switchable protein with the chromophore formed from the same amino acid residues Gly65, Tyr66, Gly67, as in Venus. Hydrogen atoms were added manually using molecular mechanics tools; the side chains of Arg and Lys were assumed as positively charged, the side chains of Glu and Asp as negatively charged. The model protein molecule was fully surrounded by explicit water molecules.

Structures of possible intermediates in the maturation reaction were optimized in QM/MM calculations. A large fraction of the chromophore-containing pocket was assigned to the QM-part. The Gly65–azF66–Gly67 fragment of the immature chromophore (CRO), the side chains from Arg96, Tyr203, Ser205, Glu222 and 4 water molecules were included. This initial composition was considered to model structures without the oxygen molecule. In majority of calculations, the O_2_ species was inserted to the cavity near CRO. Calculations of energies and energy gradients in QM were carried out using Kohn–Sham DFT with the PBE0 functional^67^ and the cc-pVDZ basis set. The AMBER force field was used in MM. The NWChem software package^68^ was applied to scan fragments of potential energy surface. These scans along with the previous experience in modelling chromophore maturation in GFP^35^ allowed us to construct protein structures of potential intermediates, which were optimized in QM/MM calculations. To model the system in the triplet electronic state, the unrestricted DFT approach was used.

Vertical excitation energies at selected points on the ground state potential energy surface were computed using the extended multiconfigurational quasi-degenerate perturbation theory in the second order (XMCQDPT2)^69^ the protocol that we verified earlier and used extensively in studies of the photoreceptor proteins.^70^ Here, the perturbation theory calculations were based on the complete active space self-consistent field (CASSCF) wavefunctions obtained by distributing 16 electrons over 12 orbitals and using density averaging over 15 states. To perform these calculations using the Firefly quantum chemistry package,^71^ large molecular clusters including the QM parts of the system were selected. Natural Bond Orbital (NBO) analysis was used to evaluate stereoelectronic interactions.^39,40^ Geometry optimizations for NBO evaluations were performed with SMD^72^ for solvation corrections and the unrestricted wB97X DFT functional^73^ (with an integration grid of pruned 175 974 for first-row atoms and 250 974 for atoms in the second and later rows) with the 6-311++G(2d,p) basis set for all atoms. Grimme's D2 version for empirical dispersion^74,75^ was also included. Natural Bond Orbital (NBO) analyses were performed with NBO6 linked to Gaussian 16. They were used to gauge the magnitude of the hyperconjugative interactions in the presented systems.

## Author contributions

All authors contributed to the writing of the paper and analysing data. HSA undertook structural biology and functional characterisation of the Venus proteins; TH helped prepare mutant proteins. OEA prepared mutant protein and generated mass spectra. PJR collected structural data and helped with structure determination and refinement, and with preparing the manuscript. BLG, IVP and AVN developed computational models and carried out computer simulations. IA and GPG provided analysis of stereoelectronic factors involved in the formation and reactivity of hydroperoxide intermediates. CB analysed data. DDJ conceived and directed the project, and contributed to data analysis.

## Conflicts of interest

There are no conflicts to declare.

## Supplementary Material

SC-012-D0SC06693A-s001
