# Supplementary material for: Stalling chromophore synthesis of the fluorescent protein Venus reveals the molecular basis of the final oxidation step
Source: Chem Sci. 2021 Mar 31;12(22):7735–45. doi: 10.1039/d0sc06693a (PMC8188506; doi:10.1039/d0sc06693a)
Supplement: SC-012-D0SC06693A-s001 [file SC-012-D0SC06693A-s001.pdf]

Electronic Supplementary Material (ESI) for Chemical Science.
This journal is © The Royal Society of Chemistry 2021

Supporting Information.

Supporting Methods.

Venus^{66azF} gene sequence

ATGCGGGGTTCTCATCATCATCATCATCATGGTATGGCTAGCATGACTGGTGGACAGCAAATGGGTCGGGATCTG
TACGAGAACCTGTACTTCCAGGGCTCGAGCATGGTGAGCAAGGGCGAGGAGCTGTTCACCGGGGTGGTGCCCATC
CTGGTCGAGCTGGACGGCGACGTAAACGGCCACAAGTTCAGCGTGTCCGGCGAGGGCGAGGGCGATGCCACCTAC
GGCAAGCTGACCCTGAAGCTGATCTGCACCACCGGCAAGCTGCCCGTGCCCTGGCCCACCCTCGTGACCACCCTG
GGC**TAG**GGCCTGCAGTGCTTCGCCCGCTACCCCGACCACATGAAGCAGCACGACTTCTTCAAGTCCGCCATGCCC
GAAGGCTACGTCCAGGAGCGCACCATCTTCTTCAAGGACGACGGCAACTACAAGACCCGCGCCGAGGTGAAGTTC
GAGGGCGACACCCTGGTGAACCGCATCGAGCTGAAGGGCATCGACTTCAAGGAGGACGGCAACATCCTGGGGCAC
AAGCTGGAGTACAACTACAACAGCCACAACGTCTATATCACCGCCGACAAGCAGAAGAACGGCATCAAGGCCAAC
TTCAAGATCCGCCACAACATCGAGGACGGCGGCGTGCAGCTCGCCGACCACTACCAGCAGAACACCCCCATCGGC
GACGGCCCCGTGCTGCTGCCCGACAACCACTACCTGAGCTACCAGTCCGCCCTGAGCAAAGACCCCAACGAGAAG
CGCGATCACATGGTCCTGCTGGAGTTCGTGACCGCCGCCGGGATCACTCTCGGCATGGACGAGCTGTACAAGTAA

Engineering and production of Venus^{66azF}

The Venus plasmid was originally obtained from Addgene (Venus-pBAD, #54859). The introduction of TAG substitution in place of the codon for Tyr66 was performed using whole plasmid (or inverse) PCR with mutagenic primer Venus-TAG-66-F (5’-GGC**TAG**GGCCTGCAGTGCT-3’; the letter in bold represent the introduced TAG mutation) and Venus-TAG-66-R (5’-CAGGGTGGTCACGAGGGTG-3’). Whole plasmid PCR was performed using the gene encoding Venus resident within the pBAD plasmid as template and Q5 DNA polymerase as described previously [1]. Introduction of the mutation was confirmed by DNA sequencing. The recombinant production of azF-containing Venus variants in *E. coli* was performed as described previously [1]. The plasmid pDule-azF was (a kind gift for Ryan Mehl [2] and supplied by Addgene) was used to facilitate incorporation of exogenous azF (0.5 mM) supplemented to 2xYT culture medium. From this point onwards, all stages were performed in the dark or samples wrapped in foil to prevent premature photolysis. Production of Venus was induced by the addition of 0.2% (w/v) arabinose when the culture reached OD_{600} of 0.7. The cells were incubated a further 20 hr at 25ºC. Cells were pelleted and then resuspended in 50 mM Tris-HCl, pH 8 followed by lysis using a French pressure cell press. Venus was purified initially by nickel affinity chromatography using a gravity flow Protino^{R} Ni-TED 2000 affinity column. Venus

was eluted using 50 mM Tris-HCl, 250 mM imidazole, pH 8. Pure protein fractions were pooled and polished by size exclusion chromatography using a Hiload^{TM} 16/600 Superdex^{TM} S75 column equilibrated with 50 mM Tris-HCl, pH 8.

Structure determination of Venus^{66azF}

For dark state Venus^{azF66} sample, a concentrated purified sample (~20 mg/ml) was used for screening crystal formation using the PACT *premier*^{TM} HT-96 screen and JCSG-plus^{TM} HT-96 screen (Molecular Dimensions, UK). The sitting drop vapour diffusion method was used in a sealed tray and kept at 20°C. All the steps of crystal preparation were performed in the dark. The growth of crystals was monitored under a light microscope, and grown crystals were harvested by picking individual crystals in a mounted lithololoop (Molecular Dimensions) and plunging them into liquid nitrogen. X-ray diffraction collection, analysis and structure determination are outlined in the main text.

Bacterial live cell imaging.

Widefield fluorescence microscopy was used for bacterial live cell imaging. For photoactivation of Venus^{Y66azF}, *E. coli* Top10 cells were first induced to express Venus^{66azF}. A Coverwell^{TM} imaging chamber (Sigma-Aldrich) was fixed on a glass slide and 0.2 ml of 1% agarose was applied before the induced bacterial cells (0.2 ml) were added and covered with a cover slide. A sample of uninduced bacterial cells was prepared in the same manner as a control. Slides for induced and uninduced bacterial cells were exposed to UV-light for 1 min at a distance of about 1 cm. Transmitted and widefield fluorescence images visualised using an inverted Olympus IX73 widefield fluorescence microscope. Images were collected with a Hamamatsu Orca flash 4.0 camera with x100 objective lens using HCImaging software and a Prior Lumen200Pro light source. Fluorescence emission was separated by a multiband dichroic emission filter set 69002 (Chroma), a wavelength of 450 nm was used for the excitation.

Mass spectrometry.

Protein liquid chromatography-mass spectrometry (LC-MS) was acquired on a Waters Acquity H-Class UPLC system coupled to a Waters Synapt G2-Si

quadrupole time of flight mass spectrometer. The column used was a Waters Acquity UPLC Protein C4 BEH column 300 Å, 1.7 µm (2.1 x 100 mm) held at 60 °C with samples elutes over an acetonitrile/water gradient containing 0.2% formic acid. The flow rate was 0.2 mL/min. Mass spectrometry data was collected in positive electrospray ionisation mode and the data analysed using Waters MassLynx 4.1. Deconvoluted mass spectra were generate using the MaxEnt1 software.

Supporting Table S1. Spectral characteristics of fluorescent proteins.

Variant	λ_{max} (nm)		λ_{em} (nm)		ε ($M^{-1}cm^{-1}$)		QY	
	Dark	UV	Dark	UV	Dark	UV	Dark	UV
[a] Venus^{WT}	515	N/A	528	N/A	92200	N/A	0.45	N/A
Venus^{66azF}	352	440	N/A	511	30260	18500	N/A	0.1
[b] sfGFP^{66azF}	391	440[c]	500	500	42870	24620	0.13 d	0.16

[a] data from [1]; [b] data from [3]; [c] final chromophore maturation product is the phenyl amine; [d] excitation at 446 nm, where ε is 2300 $M^{-1}cm^{-1}$

Supporting Table S2. Crystal structure diffraction and refined statistics of dark state Venus^{66azF}

PDB Code	6SM0
**Data collection/reduction statistics [a]**	
Diamond Beamline	I04-1
Wavelength	0.91587
pH	5.0
Crystallisation Condition	0.01 M Zinc chloride, 0.1 M Sodium acetate, 20% w/v PEG 6000
a, b, *c* (Å)	50.947, 63.251, 69.915
Space group	$P\ 2_1\ 2_1\ 2_1$
Resolution (Å)	1.909 – 63.26
Outer shell	1.909 – 1.96
R-merge (%)	16.4 (114.5)
R-pim	7.5 (70.1)
R-meas (%)	18.1 (135.3)
CC1/2	0.989 (0.355)
I / σ	7.4 (1.8)
Completeness (%)	99.9 (99.9)
Multiplicity	5.7 (3.7)
Total Measurements	103,864 (4,920)
Unique Reflections	18,146 (1,317)
Wilson B-factor(Å^2)	16.0
**Refinement Statistics**	
Non-H Atoms	2,118
Chromophore non-H atoms	21
R-work reflections	17,175
R-free reflections	1,238
R-work/R-free	19.4 / 24.2
**rms deviations (Maximum Likelihood targets in brackets)**	
Bond lengths (Å)	0.013
Bond Angles (°)	1.854
Coordinate error [b]	0.135
Mean B value (Å2)	19.6
Mean B value (Å^2) chromophore	20.4
**Ramachandran Statistics**	
Favoured/allowed/Outliers	187 / 5 / 0
%	97.4 / 2.6 / 0.0

[a] Figures in brackets refer to outer resolution shell, where applicable.
[b] Coordinate Estimated Standard Uncertainty in (Å), calculated based on maximum likelihood statistics.

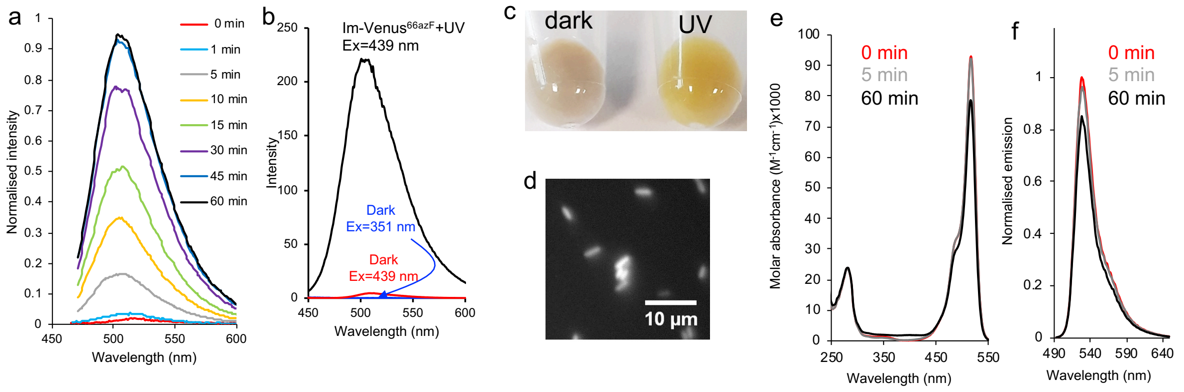

Supporting Figure S1. (a) Time course of Venus^{66azF} fluorescence emission (on excitation at 435 nm) on irradiation for different time points indicated in the figure. (b) Emission profile of im-Venus^{66azF} on excitation at 351 nm (blue) and 439 nm (red) before UV exposure and emission on excitation at 439 nm after UV exposure (black). (c and d) *in situ* activation of Venus^{66azF} with *E. coli* cell cultures before and after UV irradiation (c) and wide-field imaging of *E. coli* cells after activation with UV (d). The effect of UV irradiation on the (e) absorbance and (f) fluorescence Venus^{WT}. Irradiation was performed for the timescales indicated in figure.

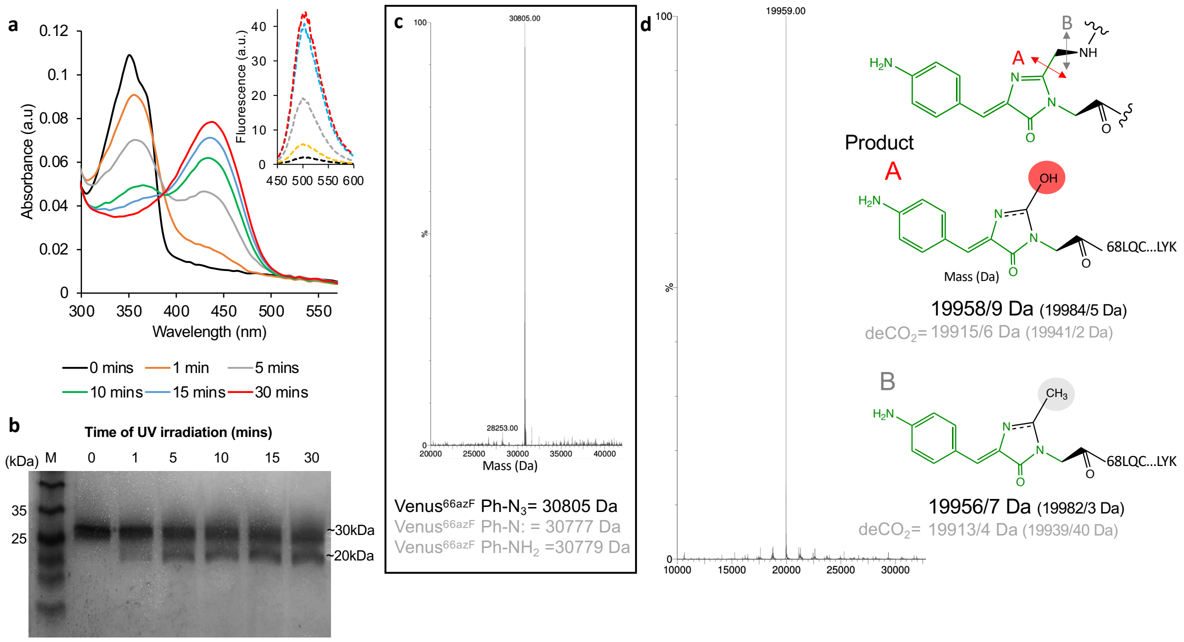

Supporting Figure 2 Mass analysis of Venus^{66azF}. (a) UV-vis absorbance spectra of Venus^{66azF} (5 μM) irradiated for the times shown in the figure. (b) Corresponding SDS-PAGE analysis of the irradiated samples. It appears that the photolyzed form is prone to fragmentation. (c-d) Electrospray ionisation time-of-flight (ESI-ToF) analysis of Venus^{66azF} (c) before irradiation and (d) after 30 min irradiation. Below each spectrum are the predicted masses for various species. (c) Three predicted masses are shown for the cyclised, dehydrated chromophore with the phenyl azide intact (black text), in the nitrene form (grey text) and phenyl-amine form (grey text). The mass corresponds to the phenyl azide form. (d) Major mass fragment of 30 min irradiated Venus^{66azF}. The mass corresponds to cleavage between the I ring and N-terminal segment of Venus^{66azF}; cleavage is proposed to occur either between C_{65}-

$C_{\alpha 65}$ bond (A) or N_{65}-$C_{\alpha 65}$ (B). Masses for a single C-N or double C=N forms are shown separated by a /. Masses for the phenyl amine are shown with the mass of the phenyl azide in brackets. Mass of a single decarboxylation of a glutamate are also shown in grey text. The mass is closest to the phenyl amine version of product A (hydroxylated product of C_{65}-$C_{\alpha 65}$ cleavage although we cannot rule out product B (methylated product cleavage between N_{65}-$C_{\alpha 65}$). All masses where calculated using the ExPasy Compute pI/Mw tool (https://web.expasy.org/compute_pi) and modified appropriately for post-translational modifications.

Supporting Figure 2. Discussion. *No irradiation.* The mass analysis of the sample prior to irradiation provides additional evidence that Venus^{66azF} has undergone dehydration and retains the phenyl azide group in the chromophore. If hydration had not taken place, we would expect to see mass peaks of 16-18 Da higher than that observed. *Irradiation.* On irradiation, the samples of Venus^{66azF} used here replicated the data shown by samples used to generate data in the main manuscript. After 1 min UV irradiation, Venus^{66azF} transform to an intermediate that directly converts to the 440 nm absorbing species (isobestic point ~390 nm). We then followed the reaction by SDS-PAGE and found a fragment at ~20kDa appeared after 1 min irradiation. The strength of this band remained relatively constant between 5-30 mins (20-25% as based on densitometry analysis using ImageJ). Given that we still see absorbance changes and fluorescence increase between 1-30 mins, we think that fragmentation is not a product of photolysis but the more commonly observed, denaturation/unfolding induced cleavage at the chromophore (Ref [4] and references therein). It does appear that the product after photolysis is more prone to fragmentation. This could be due to conversion to the phenyl amine, which can generate resonant forms that in turn may promote cleavage between the proposed C_{65}-$C_{\alpha 65}$ bond that will become solvent accessible once denatured. Under the conditions of the ESI-ToF (water/acetonitrile mix with 0.2% formic acid at 60°C), the fragmentation appears more extensive than under SDS-PAGE, with the major product being 19958-19959 Da. It is known that harsher denaturation conditions can promote fluorescent protein fragmentation (Ref [4] and references therein). The major product equates to conversion of the phenyl azide to the phenyl amine and cleavage between C_{65}-$C_{\alpha 65}$ (see Figure S2d for proposed structures) to generate a hydroxylated product (Product A). We cannot fully rule out a methylated product (Product B) potentially due to cleavage between N_{65}-$C_{\alpha 65}$ but Product A is more likely to result from a tentative hydrolysis reaction. There is precedence that such fragmentation products of denatured GFP-derived proteins with modified chromophore compositions can occur as demonstrated by Getzoff and colleagues [5] using protein X-ray crystallography. There is no evidence that additional covalent changes have occurred in the fragment, such as UV induced decarboxylation of Glu222 reported previously [6]. Thus, we believe that fragmentation is due to exposing the phenyl amine version of Venus^{66azF} to denaturing conditions so promoting cleavage between C_{65}-$C_{\alpha 65}$ rather than any UV induced cleavage events.

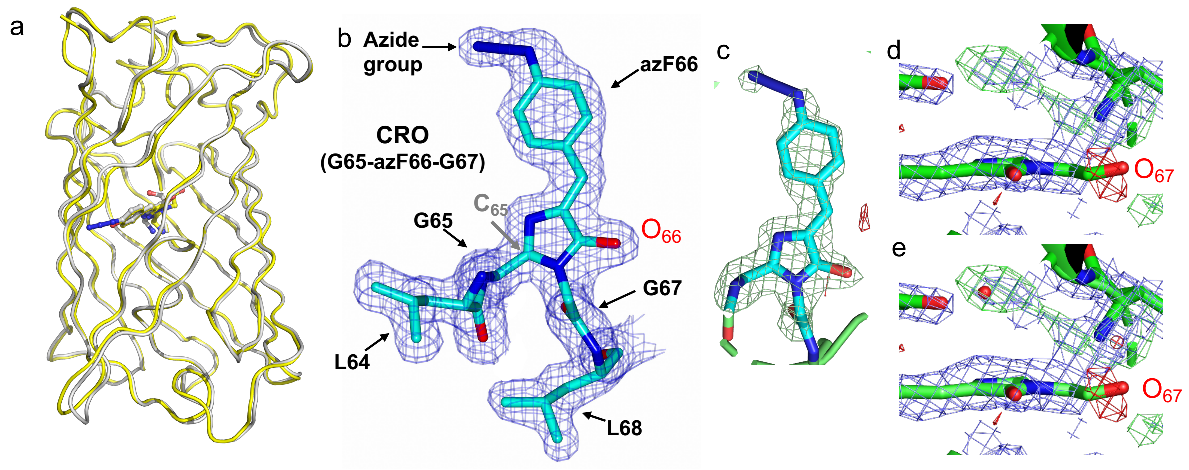

Supporting Figure S3. (a) Overlay of the Venus^{WT} (yellow) and im-Venus^{66azF} (grey). The chromophore is shown as sticks. The RMSD between backbone is 0.39 Å. Chromophore structure of im-Venus^{66azF}. (b) Electron density map (2Fo-Fc, 1.0 sigma) of the CRO. Each residue component of the original sequence is shown. The C_{65} carbon where a hydroxyl group would be observed prior to the dehydration step is outlined; no electron density for the expected hydroxyl group is observed. The C_{66}-O_{66} bond indicated by the O66 annotation, is consistent with a single bond at 1.48Å. (c) The final coordinates of the chromophore superposed on the omit map in that region. Positive difference density contoured at +3σ, negative difference density contoured at -3σ. (d-e) Omit map density near the chromophore, in the early stages of REFMAC refinement before including nonprotein atoms. Observed density (blue-grey) contoured at 1σ, positive difference at +3σ (green), and negative difference at -3σ (red). Automatic placement of waters by COOT in this map located a single water molecule at the site later described as an O_2 molecule. Note the excess density beyond the water site, which persisted throughout refinement until replaced by an O2 molecule (Figure 2, main manuscript). Also, note G67 in the canonical C_{67}-O_{67} conformation observed for wild-type Venus results in negative density, which is resolved through formation of the non-canonical twisted arrangement shown in Figure S3b and Figure 2 in the main manuscript.

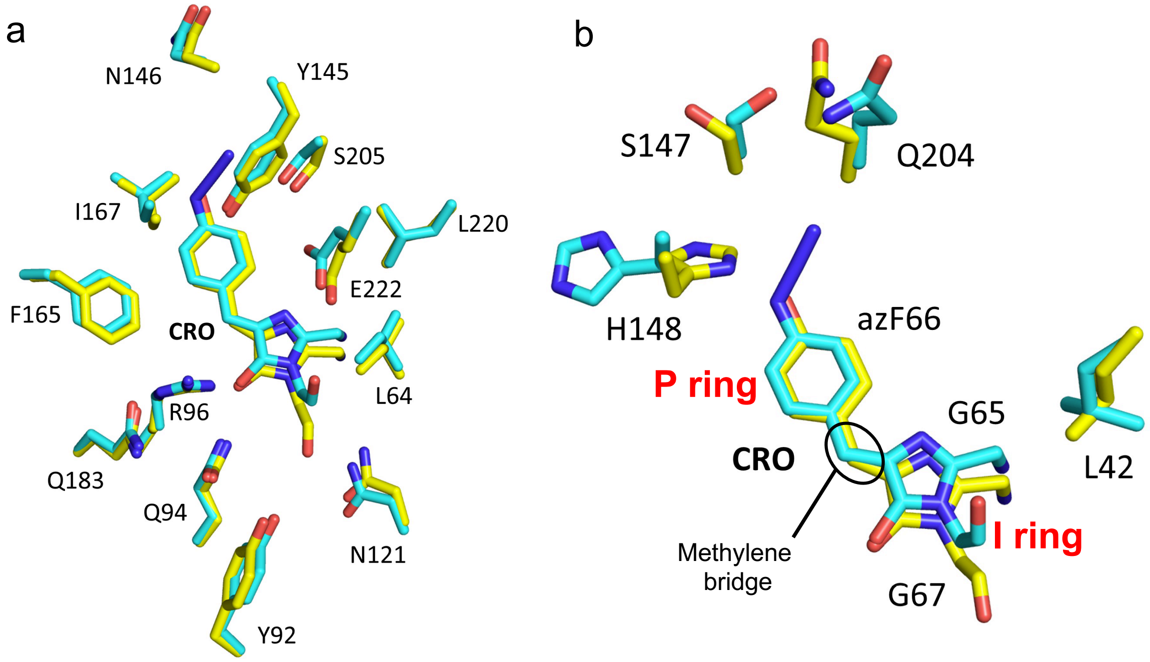

Supporting Figure S4. Comparison of residue positions between Venus^{WT} (yellow) and im-Venus^{66azF} (cyan). Panels a and b show different residue sets for clarity, with residues undergoing a larger conformational change highlighted in b.

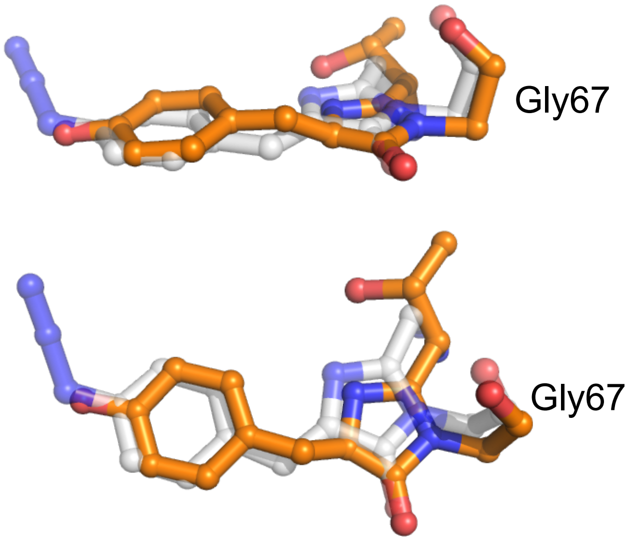

Supporting Figure S5. Comparison of the chromophore structure of im-Venus^{66azF} (grey) and unpublished GFP mutant Q183E (PDB 2qt2; orange). The GFP mutant has a partially formed chromophore and the same orientation of the Gly67 carbonyl oxygen observed for im-Venus^{66azF}.

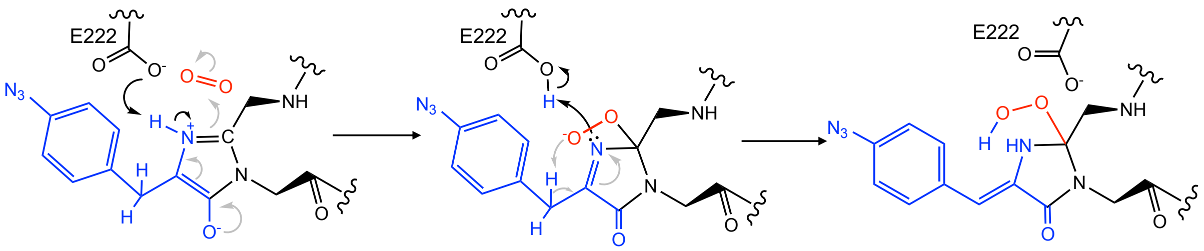

Figure S6. Schematic outline of the reaction of O_2 with im-Venus[66azF], including the potential intermediates and the role of residue E222.

Supporting References

1. H. L. Worthy, H. S. Auhim, W. D. Jamieson, J. R. Pope, A. Wall, R. Batchelor, R. L. Johnson, D. W. Watkins, P. Rizkallah, O. K. Castell and D. D. Jones, *Communications Chemistry*, 2019, **2**, 83.
2. S. J. Miyake-Stoner, C. A. Refakis, J. T. Hammill, H. Lusic, J. L. Hazen, A. Deiters and R. A. Mehl, *Biochemistry*, 2010, **49**, 1667-1677.
3. S. C. Reddington, P. J. Rizkallah, P. D. Watson, R. Pearson, E. M. Tippmann and D. D. Jones, *Angew Chem Int Ed Engl*, 2013, **52**, 5974-5977.
4. J. Wei, J. S. Gibbs, H. D. Hickman, S. S. Cush, J. R. Bennink and J. W. Yewdell, *J Biol Chem*, 2015, **290**, 16431-16439.
5. D. P. Barondeau, C. J. Kassmann, J. A. Tainer and E. D. Getzoff, *J Am Chem Soc*, 2006, **128**, 4685-4693.
6. J. J. van Thor, T. Gensch, K. J. Hellingwerf and L. N. Johnson, *Nat Struct Biol*, 2002, **9**, 37-41.
